# Microfluidic droplets with amended culture media cultivate a greater diversity of soil microorganisms

**DOI:** 10.1128/aem.01794-24

**Published:** 2025-02-12

**Authors:** Jing Dai, Yang Ouyang, Rohit Gupte, Xiao Jun A. Liu, Yuwen Li, Fang Yang, Shaorong Chen, Tony Provin, Erin Van Schaik, James E. Samuel, Arul Jayaraman, Aifen Zhou, Paul de Figueiredo, Jizhong Zhou, Arum Han

**Affiliations:** 1 Department of Electrical and Computer Engineering, Texas A&M University 189673 , College Station, Texas, USA; 2 Institute for Environmental Genomics, University of Oklahoma 6187 , Norman, Oklahoma, USA; 3 Department of Biomedical Engineering, Texas A&M University 549134 , College Station, Texas, USA; 4 Department of Chemical Engineering, Texas A&M University 14736 , College Station, Texas, USA; 5 Department of Microbial Pathogenesis and Immunology, Texas A&M University Health Sciences Center 12333 , Bryan, Texas, USA; 6 Department of Soil and Crop Sciences, Texas A&M University 199056 , College Station, Texas, USA; 7 Christopher S Bond Life Sciences Center, University of Missouri 124571 , Columbia, Missouri, USA; 8 Department of Molecular Microbiology and Immunology, University of Missouri School of Medicine 12271 , Columbia, Missouri, USA; 9 Department of Veterinary Pathobiology, University of Missouri School of Veterinary Medicine 219018 , Columbia, Missouri, USA; 10 School of Biological Sciences, University of Oklahoma 6187 , Norman, Oklahoma, USA; 11 School of Civil Engineering and Environmental Sciences, University of Oklahoma 6187 , Norman, Oklahoma, USA; 12 School of Computer Sciences, University of Oklahoma 6187 , Norman, Oklahoma, USA; 13 Earth and Environmental Sciences, Lawrence Berkeley National Laboratory 1666 , Berkeley, California, USA; Georgia Institute of Technology, Atlanta, Georgia, USA

**Keywords:** soil microorganisms, microorganism diversity, soil extracts, soil metabolites, droplet microfluidics, single-cell-resolution cultivation

## Abstract

Uncultivated but abundant soil microorganisms have untapped potential for producing broad ranges of natural products, as well as for bioremediation. However, cultivating soil microorganisms while maintaining a broad microorganism diversity to enable phenotyping and functional analysis of as diverse individual isolates as possible remains challenging. In this study, we developed and tested the ability of several culture media formulations that contain defined soil metabolites or soil extracts to maintain microorganism diversity during culture. We also assessed their performance in microfluidic droplet cultivation where single-soil microorganism isolates were encapsulated and cultivated in picoliter-volume water-in-oil emulsion droplets to enable clonal growth needed for downstream functional analyses. Our results show that droplet cultivation with media supplemented by soil extract or soil metabolites enables the recovery of soil microorganisms with higher diversity (up to 1.5-fold higher richness) compared to bulk cultivation methods. Importantly, 1.7-fold more of less abundant (<1%) phyla and 11-fold more of unique genera were recovered, demonstrating the utility of this method for interrogating highly diverse soil microorganisms for broad ranges of applications.

**IMPORTANCE:**

Although soil microorganisms hold a significant value in bioproduction and bioremediation, only a small fraction—less than 1%—can be cultured under specific media and cultivation conditions. This indicates that there are ample opportunities in harvesting the diverse environmental microorganisms if isolating and recovering these uncultured microorganisms are possible. This paper presents a new cultivation technique composed of isolating single-soil microorganism cell from an *in situ* soil microorganism community in microfluidic droplets and conducting in-droplet cultivation in media supplemented by soil extract or soil metabolites. This method enables the recovery of a broader diversity of the original microorganism community, laying the groundwork for a high-throughput phenotyping of these diverse microorganisms from their natural habitats.

## INTRODUCTION

Soil microorganisms are rich sources of high-value natural products, including pharmaceuticals (1), nutraceuticals (2), and biochemicals (3). They are also major decomposers of materials (4) and have the capabilities to convert molecules from one form to another, such as degrading plastic and upconverting degraded products into value-added products (5). Metagenomic analyses show that the diversity of these soil microorganisms is extremely high (6). However, most of the information on their functions is either derived from well-studied culturable microorganisms or inferred from analyses of reconstructed genomes for enzyme activities found in cultivatable counterparts (7). This is, at least in part, due to the fact that less than 1% of soil microorganisms has been cultured using conventional methods (8). Thus, to tap into the potential of vast numbers of natural products and enzymes produced by these diverse microorganisms, as well as to better understand the microorganisms that produce them, better experimental approaches to isolate, cultivate, and characterize these soil microorganisms are needed.

Traditional bulk cultivation methods, such as broth culture and solid media plate culture, have been modified to cultivate new species from difficult-to-culture soil communities. These modifications included alterations to growth medium components and physicochemical parameters, such as pH, temperature, and gas composition. In addition, various inhibitors and co-culture conditions have been tested (7, 9). However, most of these low-throughput bulk cultivation methods did not lead to a significant increase in diversity of culturable microorganisms owing to the culture environment being vastly different from the natural environment. Moreover, these bulk approaches often favor the cultivation of abundant and fast-growing microorganisms, where rare and slow-growing microorganisms were often missed. Recently, several innovative cultivation methods have been developed to cultivate a more diverse range of soil microorganisms at single clonal resolution, with the aim of identifying previously unknown or uncultivated microorganisms (7). These methods utilized a membrane diffusion-based approach (10–14) that supports *in situ* co-cultivation of microorganisms in the natural environment (14). However, these methods either remain low throughput where only hundreds to thousands of clonal populations can be recovered in each experiment or lack the capability to culture isolated single cells in their natural environments, and, therefore, were able to recover only relatively low levels of microorganism diversity.

In recent years, several microfluidic-based cultivation methods have emerged with the capability of a higher throughput than conventional approaches. Microfluidics-based microwells (e.g., slip chip [15, 16] and microchambers [16]) enabled throughputs of up to a few thousand cells per device. The high-throughput nature of droplet microfluidics is especially well suited for the aforementioned applications, where cells of different species and strains can be encapsulated and compartmentalized into picoliter-volume droplets, which enable isolation and cultivation of strains at a single-cell level. In droplet microfluidics formats, the competition between species/strains is limited, as single cells are confined within their own droplets, but communication through chemical diffusion between the multi-species/strains communities can still be achieved. It has been demonstrated that microorganisms with a higher microorganism diversity can be recovered by droplet-based cultivation techniques compared to traditional culture techniques (17, 18). More importantly, the number of simultaneous cultures can be tremendously increased, as droplets can be generated and processed at a much higher throughput, for example, at a processing speed of 10^4^ cells/min (19). This unique feature of droplet-based cultivation methods enabled the discovery of soil microorganisms that produce new antimicrobial products (17) and antibiotic-resistant species from the human microbiome (20).

Among the droplet microfluidics-based methods, agarose gels have been used to encapsulate and immobilize single microorganism cells (21, 22). The porous structure of the gel matrix allows the transport of nutrients and metabolites between encapsulated cells. The agarose gel droplets can also be placed in the environment from which the microorganisms were originally harvested. These aspects can promote the survival and growth of individual cells within those gel droplets (21). In another example of the use of droplet microfluidics, encapsulation of microorganisms in droplets containing aqueous culture media also has been demonstrated to better preserve the microorganism diversity (17, 18).

One of the important factors influencing the cultivability of environmental microorganisms is the presence of metabolites or molecules in their native habitats. Therefore, designing new culture media that mimic their favorable growth condition is thought to be critical in cultivating microorganisms without lowering their diversity for downstream phenotyping and functional characterization. Currently, there are two approaches to construct such culture media: 1) develop environmentally relevant defined media by supplementing them with metabolites present in the soil samples from which the soil microorganisms came; and 2) directly extracting organic and inorganic components from soil from which the soil microorganisms came (23). Although both droplet-based cultivation method (17, 18) and culture media amendment-based method (9) have been proven to be effective in preserving microorganism diversity, to date, there has been a lack of systematic study about whether the combination of both methods can work synergically, and, if yes, how effective they are in preserving microorganism diversity, especially in soil microorganisms.

In this study, we constructed several culture media that contained either defined soil metabolites or soil extracts. We first compared them to traditional cultivation media under bulk cultivation conditions. Next, soil microorganism cells were encapsulated in water-in-oil emulsion droplets containing the developed culture media at a single-cell resolution using a microfluidic droplet generator, and then the cell-encapsulated droplets were cultivated for up to 14 days, followed by comparing their diversities. This work paves the way for being able to interrogate a much higher diversity of soil microorganism cells in downstream high-throughput phenotypic analyses for a variety of microbiological applications, including, for example, the discovery of soil microorganism species that can decompose materials (e.g., plastics) or produce antifungal/antibacterial compounds.

## MATERIALS AND METHODS

### Soil sample collection and handling

The surface soil sample (loam, 0–10 cm) was taken from a location at 34°58′45″ N, 97°31′15″ W outside of a long-term warming site in central Oklahoma established in 2009 (24). The soil was sampled in two seasons: spring and fall. The soil was loam with a pH of 7.4. Soil organic carbon and total nitrogen were 1.23 and 0.11%, respectively. The shovel used for taking the soil sample was sterilized with 70% alcohol before sampling, and about 1.0 kg of soil was collected. The soil sample was sealed in a Ziplock bag and stored on ice during transportation back to the laboratory. The soil sample was homogenized, passed through sterilized 2 mm sieves, and stored at 4°C for a maximum of 7 days prior to cell extraction.

### Soil microorganism extraction

Soil microorganisms were extracted using our recently developed Nycodenz density gradient medium-based method (25). Nycodenz is 5-(N-2,3-dihydroxypropylacetamido)−2,4,6-tri-iodo-N,N′-bis (2,3-dihydroxypropyl) isophthalamide. It is a non-ionic, tri-iodinated derivative of benzoic acid with three aliphatic hydrophilic side chains. It has high solubility in water due to the hydrophilic groups linked to its tri-iodobenzene ring and has very low toxicity derived from the dihydroxypropylacetamido side chain, making it suitable for the gradient separation of cells, organelles, and virus. Nycodenz density gradient centrifugation is one of the most commonly used purification methods. In brief, soil was added into the PBS buffer containing 0.5% Tween 20 and dispersed by blending in a Waring blender (Conair 7012S, cat. 14-509-7G), followed by Nycodenz (80%) purification, in which the mixture was centrifuged, and the layer containing cells above the Nycodenz layer was collected. The microorganism cell pellet was then re-suspended in the PBS buffer before being cultivated.

### Soil metabolite extraction and analysis

One gram of soil was suspended in a centrifuge tube containing 4 mL of deionized water and placed on a shaker for 45 min, sonicated for 2 min, and centrifuged at 15,000 × *g* for 30 min. The supernatant was then filtered using a 0.2 µm filter. Conventional gas chromatography–mass spectrometry (GC–MS) method was performed by Agilent 6890 GC–MS (Agilent) to analyze the metabolites in the supernatant. Analysis of the GC–MS spectra was performed with the MassHunter software (Agilent), and the 2017 version of the NIST Mass Spectral Library (NIST 17) was used for the identification of metabolites. Ten most common soil metabolites from the soils, which were sampled in spring and fall seasons as described in “Soil sample collection and handling,” above, were determined as key metabolites to be supplemented to construct the synthetic culture media.

### Batch culture with traditional and amended media

Four traditional culture media [International *Streptomyces* Project medium (ISP2), Luria–Bertani medium (LB), tryptic soy broth (TSB), and Reasoner’s 2A medium (R2A)] were prepared by following the suppliers’ protocols. A nutrient-poor synthetic groundwater medium (SGW) was modified based on the work presented by Green et al. (26), which contained the following components: NaCl (1.7 mM), NH_4_Cl (1.87 mM), KH_2_PO_4_ (0.33 mM), KCl (0.14 mM), MgCl_2_·6H_2_O (0.2 mM), CaCl_2_ (0.33 mM), glucose (10 mM), yeast extract (0.1%), piperazine-N,N′-bis(ethanesulfonic acid) (PIPES, 30 mM), trace mineral solution containing (per liter of distilled water) 12.8 g nitrilotriacetic acid brought to pH 6.5 with NaOH, 1 g FeCl_2_·4H_2_O, 0.5 g MnCl_2_·4H_2_O, 0.3 g CoCl_2_·6H_2_O, 0.2 g ZnCl_2_, 50 mg Na_2_MoO_4_·2H_2_O, and 20 mg H_3_BO_3_, and vitamin solution containing (per liter of distilled water) 2 mg biotin, 2 mg folic acid, 10 mg pyridoxine hydrochloride, 5 mg thiamin hydrochloride, 5 mg riboflavin, 5 mg nicotinic acid, 5 mg dl-pantothenic acid (calcium salt), 0.1 mg vitamin B_12_, 5 mg *p*-aminobenzoic acid, 5 mg lipoic acid, and 200 mg choline chloride (27, 28). Two amended media were prepared by mixing R2A with soil extract, which was prepared as described in “Soil metabolite extraction and analysis,” above, and also by mixing R2A with the 10 identified key soil metabolites. In all tested culture medium conditions, 3 × 10^8^ soil microorganisms were inoculated into 30 mL of culture medium in a loosely capped 45 mL conical centrifuge tube. They were then incubated at room temperature (~ 20°C) in the dark without shaking. Three replicates were included for each culture medium condition.

### Microfluidic droplet culture

Soil microorganisms were suspended in culture media, and then encapsulated in water-in-oil emulsion droplets. The cell concentration was 1 × 10^7^ cell/mL before being encapsulated into 45 µm-diameter droplets. Droplets were generated by a flow-focusing microfluidic droplet generator using Novec 7500 engineering fluid (3M) with 2% Pico-surf (Sphere Fluidics) surfactant as the oil phase and the microorganism cell suspension as the aqueous phase (29). Under these conditions, 33% of the entire droplet population is expected to contain a single cell, where the entire droplet population (λ = 1, average one cell/droplet) follows the Poisson distribution (30). A total of 8 × 10^6^ cells were encapsulated for each culture medium condition, and the droplets were collected in a loosely capped 15 mL culture tube. The droplets were then incubated at room temperature (~20°C) in the dark, same as the bulk culture condition. After cultivation, the droplets were demulsified by mixing droplets with 10% 1H, 2H, 2H-Perfluoro-1-octanol (Millipore-Sigma) in Novec 7500 engineering fluid and centrifuged at 300 × *g* for 3 min. After repeating this step at least three times, the top aqueous phase containing cultured microorganisms was transferred to a 15 mL centrifuge tube for DNA extraction.

### Live/dead cell staining and flow cytometry

To quantify the number of viable and dead cells in samples, fluorescent live/dead cell staining dye SYBR Green I and propidium iodide were used to stain the cells using the manufacturer’s protocol (31, 32). Stained cell samples were processed and quantified using an Accuri C6 flow cytometer (Becton Dickinson) (25).

### High-throughput amplicon sequencing and sequence data processing

Propidium monoazide (PMA) is a commonly used chemical to remove DNA from dead cells, so that only live cell information is captured in the sequence analyses (33, 34). All cell culture samples were mixed with PMA (50 µM final concentration) and incubated in the dark for 5 min. Samples were then exposed to a 470 nm light-emitting diode lamp for 20 min with shaking every 5 min. DNA was extracted by using a QIAGEN PowerSoil DNA Extraction Kit (Qiagen), and DNA concentration was quantified by a NanoDrop spectrophotometer (NanoDrop Technologies). A two-step PCR was used to amplify the V4 region of 16S rRNA gene (16S) with the primer pair 515F (5′-GTGCCAGCMGCCGCGGTAA-3′) and 806R (5′-GGACTACHVGGGTWTCTAAT-3′) (35). A MiSeq sequencer (Illumina) was used to sequence amplicons (2 × 150 bp). The Illumina sequences were processed and analyzed using a Galaxy-based sequence analysis pipeline (25). Briefly, high-quality sequences with lengths between 245 and 260 bp were clustered into operational taxonomic units at the 97% identity using UPARSE (36), and singletons were removed. Then, taxonomic assignment was conducted through the RDP classifier version 2.13 with a confidence cutoff of 0.5 (37). The R package, phyloseq, was used to analyze alpha diversity of soil microorganisms from the 16S sequencing data (38). After raw data processing, the retained high-quality sequences were randomly resampled to a depth of 9,645 reads per sample to enable an accurate comparison of samples with different sequencing depths. The ‘observed’ richness was calculated using the function of estimate richness to assess the different number of species.

### Statistical analyses

All statistical analyses were conducted using the Prism 9 (GraphPad) software. Student’s *t*-test was used to characterize the statistical significance between two groups of conditions with 95% confidence level.

## RESULTS AND DISCUSSION

**Fig 1:**
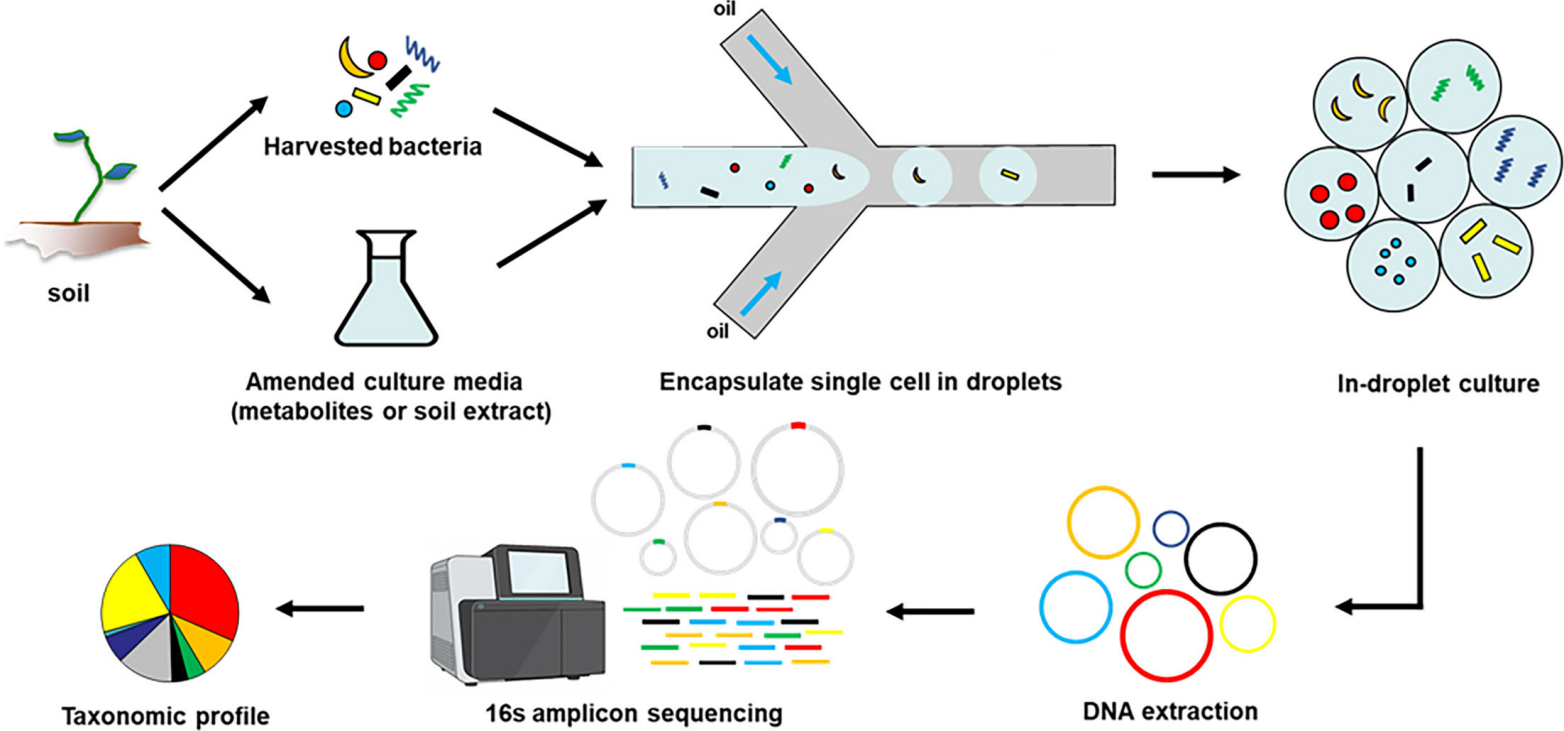
Soil microorganisms were isolated, and then encapsulated in water-in-oil emulsion droplets, together with amended culture media having key soil metabolites or soil extract, to promote clonal growth within droplets to preserve their diversity. DNA from these cell-containing droplets were extracted. 16S rRNA amplicon sequencing was used to assess the taxonomic composition of the cultured soil microorganism communities to evaluate the recovery of less-abundant taxa.

We developed a workflow that included generation and testing of soil microorganism-encapsulated droplets with different culture media to recover highly diverse microorganism communities from the soil samples (Fig. 1). In brief, soil microorganisms were extracted from the soil samples, while key metabolites or soil extracts were extracted from the soil samples to construct amended media. Next, cells were encapsulated in water-in-oil emulsion droplets at a single-cell resolution in various culture media using a microfluidic droplet generator, followed by droplet cultivation. We reasoned that the droplet-based cultivation method would prevent overgrowth of fast-growing or high-abundance strains, a phenomenon often observed in bulk culture, by isolating and separating fast-growing species into individual droplet microfluidics carriers. This will enable slow-growing or low-abundance microorganisms to be multiplied in individual droplets in the absence of competition, maintaining the overall microorganism population diversity. This strategy was evaluated by measuring community diversity using 16S rRNA amplicon sequencing, so that the taxonomic composition of the soil microorganism community could be assessed.

### Identification of a base media for amended culture media development

**Fig 2:**
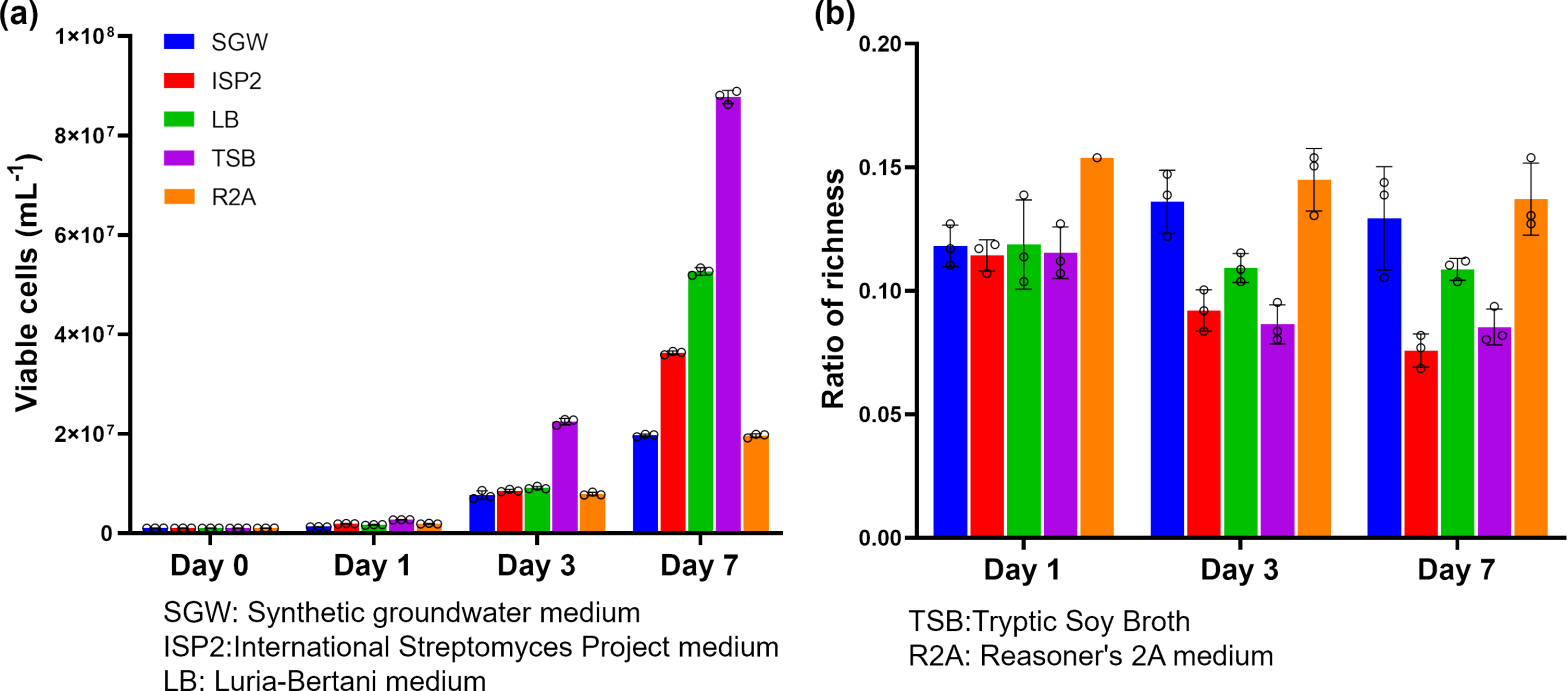
Viable cell number and richness of soil microorganisms cultured in five different traditional culture media over 7 days. (**a**) Viable cell count. (**b**) Ratio of richness (richness at days 1, 3, and 7 compared to richness at day 0). Open circles denote the three replicates of each sample.

As a first step toward developing amended culture media that can maintain microorganism diversity as much as possible, we first performed experiments to identify the base media that would maximize diversity. To identify the desired base media, we compared the diversities of microorganism communities following incubation in five commonly used environmental microorganism culture media, namely, SGW, ISP2, LB, TSB, and R2A, for 7 days at room temperature. Analysis of samples at different times indicated that all culture media supported the growth of soil microorganisms with increased viable cell counts over 7 days (Fig. 2a). On day 7, the highest increase in the viable cell count was 92-fold increase in TSB, while the lowest increase (19-fold) was in R2A (in both cases compared to the day 0 viable cell count). It is known that microorganism diversity can be simply measured in terms of richness (39). Therefore, we quantified the richness of each sample over the 7-day culture period. The results show that R2A maintained richness the most (14%, defined as the ratio between richness on the test day and richness at day 0) (Fig. 2b). R2A has relatively limited nutrient, as it was developed to limit the growth of fast-growing bacteria that can rapidly deplete nutrient and allow growth of slow-growing bacteria (40). Thus, it was expected that a higher microorganism diversity would be maintained in R2A compared to other nutrient-rich media, such as TSB, which indeed showed 5% more reduction in richness. Based on this result, R2A was chosen as the base culture medium in all subsequent experiments, where R2A was modified by supplementing it with different components to further increase the diversity of the viable soil microorganism community during cultivation.

### Amended culture media displays improved performance

**Fig 3:**
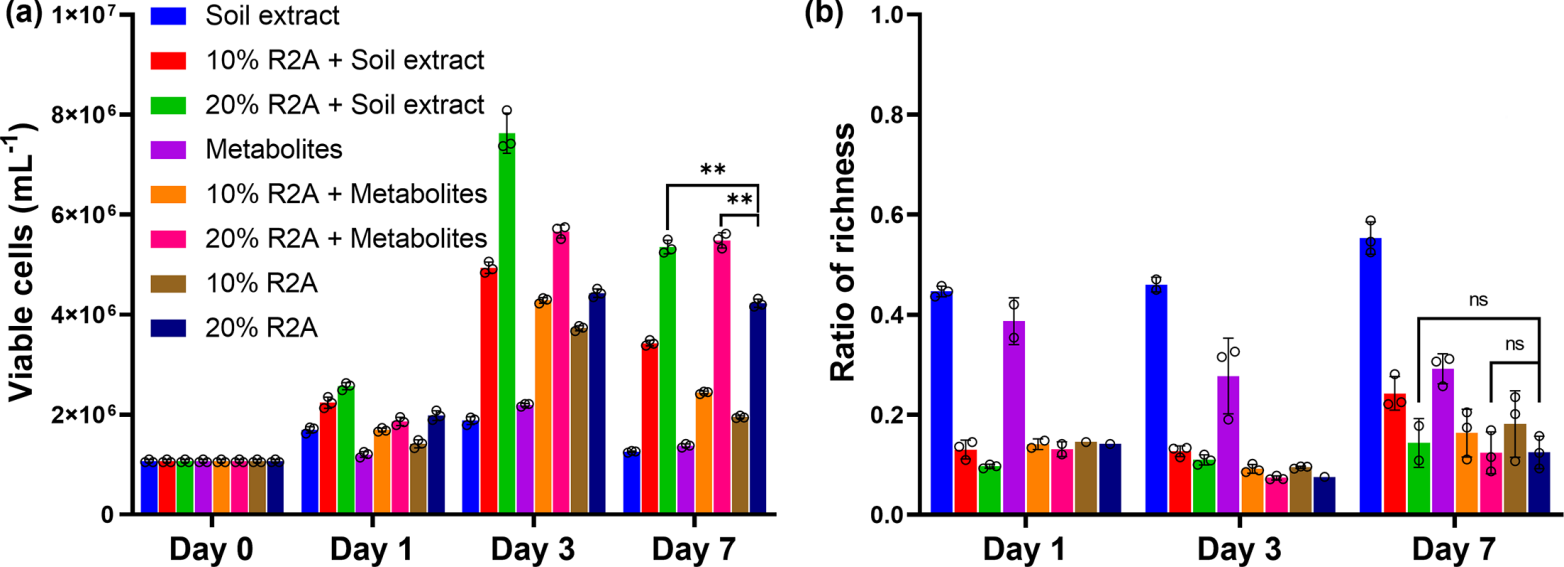
Diversity and viable cell count of soil microorganism cells cultured in seven different media over 7 days. (**a**) Viable cell count. (**b**) Ratio of richness from days 1 to 7 compared to richness at day 0. ** denotes *P* < 0.001; ns denotes non-significance. Pairwise statistical significance analysis was performed for 20% R2A against 20% R2A + soil extract and against 20% R2A + metabolites. Open circles denote the three replicates of each sample.

To optimize the culture media so that the cultured soil microorganism community could maintain higher viability and diversity, two different amended culture media were prepared: R2A base medium amended with 10 key metabolites, which are commonly presented in nine loam soil samples (Table 1), and R2A amended with soil extracts. For both cases, two different concentrations of amendments were tested. Diluted R2A (10 or 20%), as well as soil extract alone, were tested as controls (Fig. 3).

**TABLE 1:** Top 10 metabolites added to R2A to construct an amended medium

Metabolite	Concentration (mg/L)
Adenosine	1.7
Benzoic acid	0.7
Betaine	20.0
Choline	5.5
Cytosine	0.4
Guanine	0.4
Hypoxanthine	0.2
D-Proline	0.5
DL-Leucine	1.1
DL-Carnitine	3.6

When the extracted soil microorganisms were cultured in soil extract alone, 55% of the richness was retained, while the number of viable cells increased by 18% over a 7-day culture period. In comparison, 29% of richness was retained, and the viable cell count increased by 29% when the cells were cultured in 10 key metabolites alone. Although in both cases the culture maintained higher richness, their relatively low viable cell counts indicate that cell growth was also limited possibly due to the fact that soil extract is a “poor” nutrient medium (41). Thus, soil extract alone or metabolites alone are not sufficient to grow and expand soil microorganisms and are, thus, not the best choice.

R2A has a lower nutrient content compared to a rich culture medium, such as TSB or LB (41). However, R2A is still a relatively nutrient-rich medium, and it has been previously reported that, by diluting R2A (instead of using 100% R2A), a higher microorganism community richness can be maintained (42). Here, cultivation of soil-extracted microorganism communities with 10 or 20% diluted R2A indeed demonstrated similar results. On day 7, soil microorganism communities cultured in 10% R2A retained 18% of the richness, while those in 20% R2A retained 12%. However, the viable cell numbers following cultivation in 10% R2A were lower, about half of that of 20% R2A culture. This result suggests that 20% R2A is a potentially good base culture medium for amendment, where a balance between cell growth and diversity maintenance can be achieved.

Next, R2A was amended with soil extracts or key soil metabolites to investigate how they impact the richness and viability after culture. Viable cell numbers in cultures of both 20% R2A + soil extract (20% R2A + SE) and 20% R2A + metabolites (20% R2A + M) were 30% higher (in both cases) than that of 20% R2A at day 7. In both cases, microorganism community richness did drop significantly compared to the original community but without significant differences between 20% R2A and the amended media. However, this drop in richness was similar to all other culture media conditions, except for soil extract, while the viability was higher than all other culture conditions. Taken together, these data suggest that these amended culture media provide substantial benefit over their conventional counterparts and were, therefore, used to test the working hypothesis that further improvement in the recovery of soil microorganisms could be achieved by combining these insights with the droplet microfluidics-based cell growth strategy.

### Microorganism viability in droplet culture

**Fig 4:**
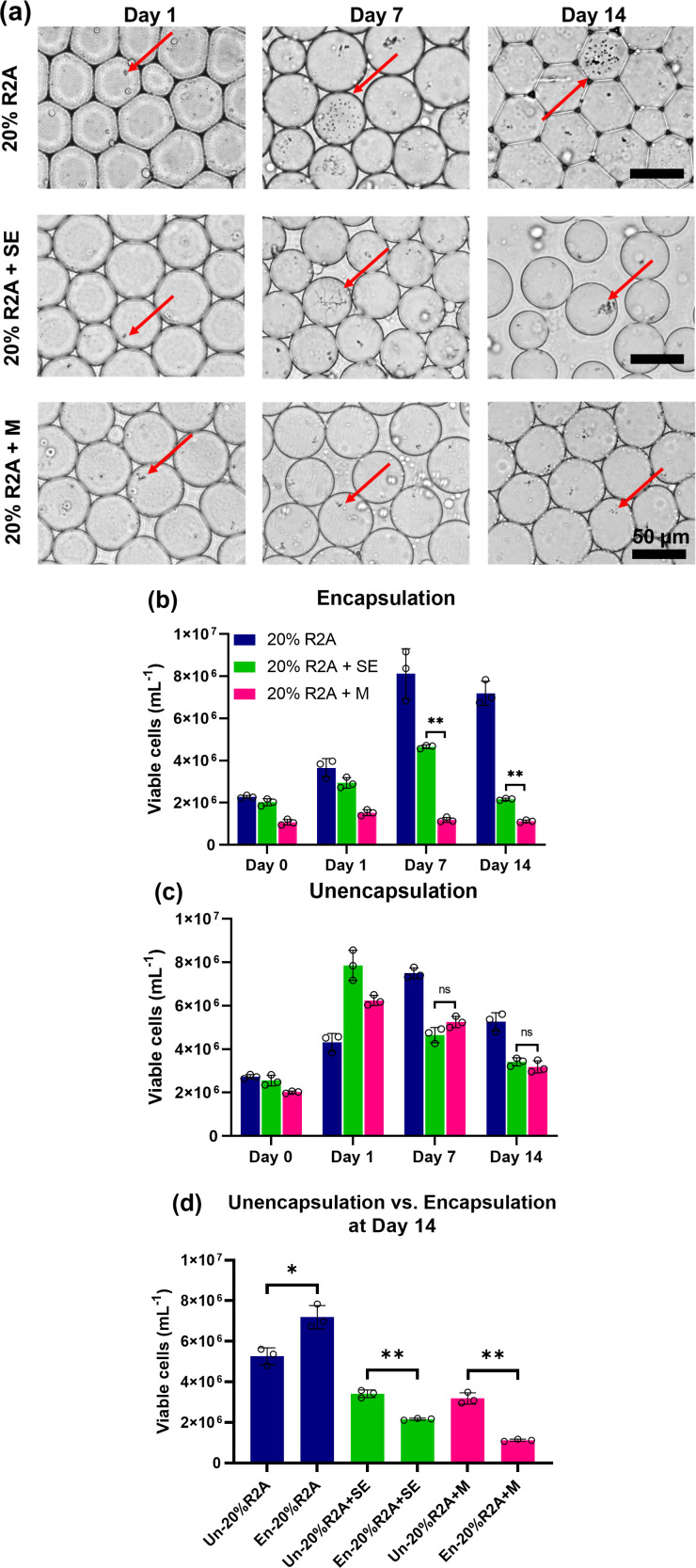
Soil microorganisms were encapsulated with three different culture media. (**a**) Time-lapsed images of cell culture in microdroplets over 14 days. Viable cell counts of (**b**) encapsulated and (**c**) unencapsulated samples. (**d**) Viable cell count of encapsulated vs. unencapsulated sample after 14-day culture. Un denotes unencapsulation; En denotes encapsulation; * denotes *P* < 0.05; ** denotes *P* < 0.001; and ns denotes non-significant. For (**b**) and (**c**), pairwise statistical significance analysis was performed for 20% R2A + soil extract against 20% R2A + metabolites. Open circles denote the three replicates of each sample.

To test the hypothesis that the viability of microorganism communities in amended culture media could be further enhanced by subsequent encapsulation and cultivation of them in picoliter water-in-oil emulsion droplets, we used a previously described droplet microfluidic system (29, 43, 44) to encapsulate single-soil microorganism cells in droplets containing 20% R2A, 20% R2A + soil extract (20% R2A + SE), and 20% R2A + soil metabolites (20% R2A + M). These cell-encapsulated droplets were then cultured for 1, 7, or 14 days at room temperature, followed by analysis of their viability and growth. To gain insight into the dynamics of microorganism growth in droplets, cells in droplets were imaged over the course of the 14-day culture (Fig. 4a). By day 7, an increased cell number in individual droplets could be observed in all culture conditions. By day 14, the highest number of droplets with extensive cell growth was observed in 20% R2A, while a smaller number of droplets showed extensive cell growth when 20% R2A was supplemented with soil extract or metabolites.

To further characterize the droplet-cultivated cells, we demulsified droplets to release them from the droplet emulsion, followed by measuring their viability and diversity. Cells cultured in droplets were released by demulsifying the droplets on days 1, 7, and 14. The viable cell counts (Fig. 4b) were consistent with the imaging results, where the viable cell count in 20% R2A supplemented with soil extract is significantly higher than that from 20% R2A supplemented with metabolites after day 7. As a control, the same batch of soil microorganism cells was cultured in conventional cell culture tubes in the same culture medium conditions, representing “unencapsulated” samples (Fig. 4c). These cultures showed similar viable cell counts and growth patterns as the encapsulated microorganisms for all culture media conditions. After 14-day culture (Fig. 4d), the growth of cells in encapsulated culture was statistically different from unencapsulated sample in 20% R2A; for 20% R2A + SE, unencapsulated sample showed more growth; and for 20% R2A + M, unencapsulated sample showed more growth. This is an interesting phenomenon, as encapsulation did not improve the cell growth in the two amended culture media, worthy of a future in-depth study.

### Droplet culture better maintains microorganism diversity

**Fig 5:**
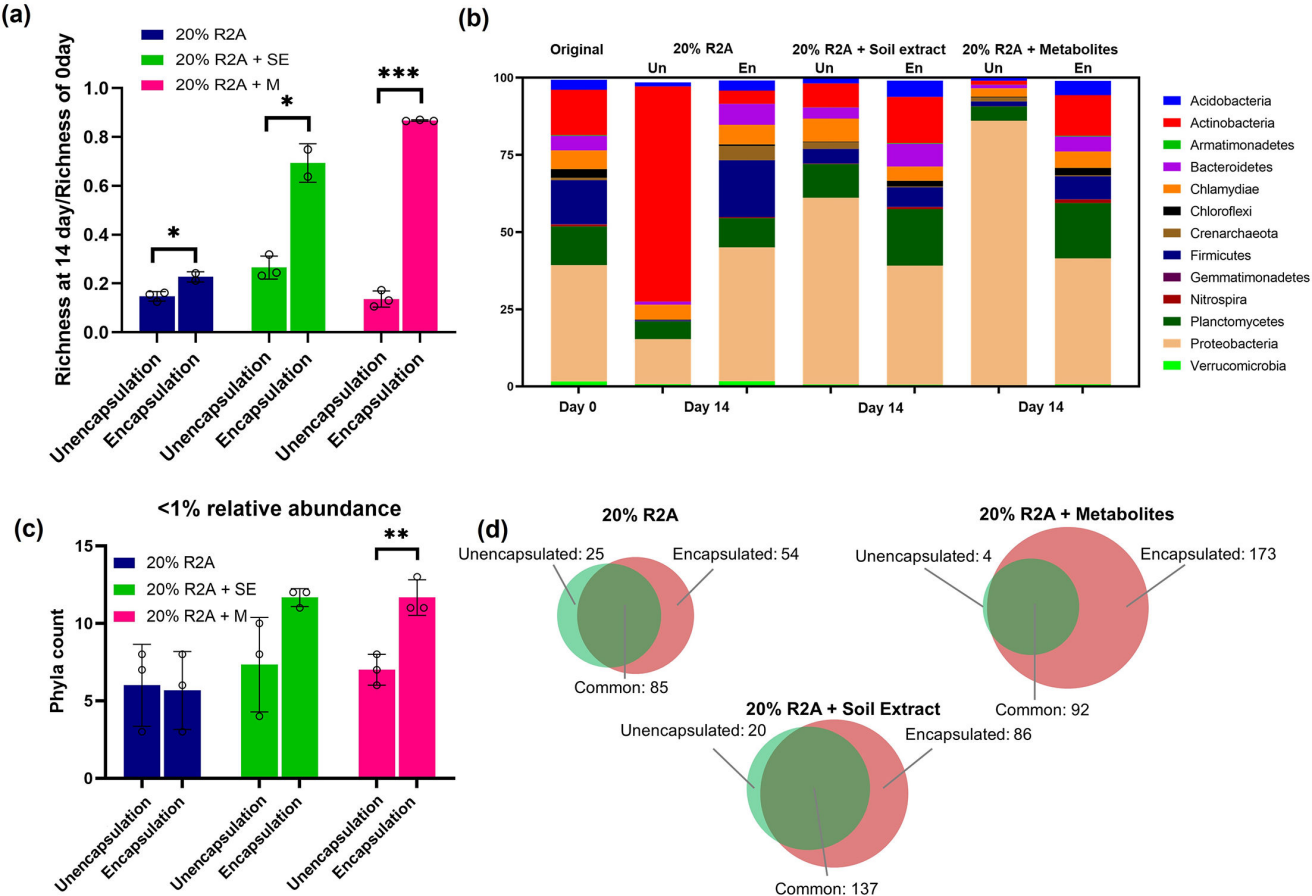
Diversity and abundance comparing unencapsulated and encapsulated soil microorganisms cultured in three different culture medium conditions after 14 days of culture. (**a**) Richness. (**b**) Relative abundance at phyla level of encapsulated (En) and unencapsulated (Un) samples. (**c**) Count of phyla with less than 1% abundance. (**d**) Venn diagram of taxa classified at genus level, excluding unclassified taxa counts, accounting for ~17% of the total operational taxonomic units. * denotes *P* < 0.05; ** denotes *P* < 0.01; and *** denotes *P* < 0.001. Open circles denote the three replicates of each sample.

We analyzed the richness and the relative abundance of both encapsulated and unencapsulated samples after 14 days of culture (Fig. 5). The richness of the two culture methods, encapsulated vs. unencapsulated, were compared and shown in Fig. 5a. In 20% R2A, 85 and 77% drops in richness were observed in unencapsulated and encapsulated culture formats, respectively. In contrast, in 20% R2A + SE, 73 and 31% drops in richness were observed in the unencapsulated and encapsulated samples, respectively. In 20% R2A + M, about 86 and 13% drops in richness were observed in the unencapsulated and encapsulated culture formats, respectively. These results show that droplet-encapsulated culture can maintain richness much better compared to conventional culture methods.

We then compared the relative abundance of the cultured communities at the phyla level between the original sample at day 0 and cultures at day 14 (Fig. 5b). A total of 13 phyla accounted for 99% abundance of the community. For all unencapsulated samples, it was observed that original diversity cannot be maintained, and a single phylum became relatively more abundant in cultures after a 14-day incubation. For example, Actinobacteria was relatively more abundant in 20% R2A, while Proteobacteria was relatively more abundant in both 20% R2A + SE and 20% R2A + M. For all encapsulated samples, the relative abundance of phyla was largely maintained after 14 days of incubation in three different media.

Previous work has shown that conventional bulk culture has the limitation that the abundant taxa are often over-represented (7). We, hence, analyzed phyla with less than 1% relative abundance in all samples (Fig. 5c). Encapsulated samples cultured in amended R2A culture media recovered more numbers of less abundant phyla (<1% abundance) in comparison to the unencapsulated sample. Specifically, encapsulated samples recovered 12 phyla, while unencapsulated samples only recovered 7. However, no significant differences in rare phyla recovered were observed between encapsulated and unencapsulated conditions under 20% R2A medium condition and 20%R2A + soil extract.

We also compared taxa classified at the genus level. A generated Venn diagram (Fig. 5d) and Table 2 show that most taxa classified at the genus level in unencapsulated culture were shared in the encapsulated culture, but encapsulated culture preserved 29, 66, and 169 more unique genera in the case of 20% R2A, 20% R2A + soil extract, and 20% R2A + metabolites, respectively. These specific taxa presented in droplet culture implies that droplet culture can preserve yet-to-be cultured taxa , which is a phenomenon also observed in prior soil microorganism culture studies (45, 46). Therefore, this result demonstrates that microfluidic droplet culture with amended R2A culture media can better maintain microorganism diversity.

**TABLE 2:** Taxa classified at genus level for both unencapsulated and encapsulated samples in three culture media conditions^*a*^

	Count and percentage of unique genera in unencapsulated sample	Count and percentage of unique genera in encapsulated sample	Count and percentage of shared genera	Total genera
20% R2A	25 (15.2%)	54 (32.9%)	85 (51.8%)	164
20% R2A + soil extract	20 (8.2%)	86 (35.4%)	137 (56.4%)	243
20% R2A + metabolites	4 (1.5%)	173 (64.3%)	92 (34.2%)	269

^*a*^ The percentage of unique genera in each sample is calculated by dividing the genera count by total genera.

In this study, we developed several amended culture media to cultivate soil microorganisms with a high-throughput droplet microfluidics method. The modification of culture media that more closely resemble their natural environment has been previously demonstrated to better preserve the diversity of cultivated microorganisms cultivated (47). We selected R2A as the base culture medium because it promoted a greater richness and viability of soil microorganisms than traditional rich culture media (Fig. 2). Since R2A is not designed to mimic the ecological environment of soil microorganisms, we hypothesized that amending R2A with metabolites and/or other substances relevant to the environmental niches from which the microorganisms came from could promote retaining of the microorganism diversity and maximize the growth of indigenous species during culture. Although soil extracts only containing minerals or organic compounds were previously shown to improve soil microorganism cultivability (48), most cultivated isolates belong to those that are also isolated from traditional culture media. It has been reported that a modified R2A culture medium for cultivating heterotrophic bacteria enables the isolation of new taxa compared to traditional media (49). Therefore, we developed culture media by amending diluted R2A with soil extract or key soil metabolites. Our results showed that these two media have maintained richness for up to 7-day culture similar to that of unamended 20% R2A (Fig. 3b) while enabling 2–9-fold higher richness than unamended 20% R2A for 14-day culture (Fig. 5a). These results demonstrate that amended 20% R2A media can better preserve soil microorganism diversity and unique taxa.

We further improved the cultivation of highly diverse soil microorganisms using a droplet microfluidics-based cultivation method to maintain their diversity. Single-soil microorganism cells were compartmentalized into millions of individual picoliter-volume droplets so that single cells could be isolated and confined within their own droplets. The addition of surfactant in continuous phase during droplet generation allowed droplets to remain stable throughout the 14-day culture (Fig. 4) while still allowing transportation of oil-soluble metabolites and signaling molecules among the encapsulated species (50). Since individual single cells were isolated and confined within each droplet, even slow-growing species will be less likely to be negatively impacted by rapid nutrition depletion by the fast-growing species, as is commonly the case in bulk cultivation. Our results showed that encapsulating single microorganism cells in droplets with amended R2A media improved their diversity, preserving low-abundance taxa at the phyla level and identifying unique taxa at the genus level at least several folds higher than unencapsulated samples (Fig. 5; Table 2). Although some recent cultivation methods have demonstrated improved recovery throughput of uncultivable soil microorganisms, such as membrane diffusion-based cultivation method (e.g., iChip)^10^ or micro-scale diffusion chambers (16), their throughput remains such that at only about a few hundred thousand potentially unique isolates to be retrieved per device. In contrast, our droplet microfluidics-based cultivation method enables processing and cultivating environmental microorganisms at a much higher throughput at least several million cells per hour while far better maintaining their diversity and viability. The isolated and clonally grown single cell can be used for downstream droplet-based high-throughput analyses of natural microorganisms for many functions, such as for bioproduction, bioremediation, and natural product discovery purposes. One example is the discovery of antifungal products, where one droplet containing an isolated single microorganism cell that has been cultured to grow into a sufficient number can be merged with another droplet containing pathogen of interest so that both species can be co-cultured. This will allow screening of environmental microorganisms that may kill or inhibit the growth of the target pathogen of interest.

### Conclusions

Obtaining viable and diverse cells representing the *in situ* microorganism community in soil as much as possible is often the first step for testing those cells for varieties of microbiological applications. Here, we constructed several culture media and found that 20% R2A culture media amended with defined soil metabolites or soil extracts can better preserve soil microorganism diversity while still supporting growth of those microorganisms. Furthermore, we applied a high-throughput droplet microfluidics-based cultivation method to isolate and culture soil microorganisms at single-cell resolution to obtain sufficient clonal population for downstream analyses. Our results indicate that combining amended 20% R2A culture media with the droplet microfluidics-based culture method contributes to maintaining the diversity and relative abundance of soil microorganisms during culture. Moreover, this method preserved less-abundant taxa and enabled the recovery of more unique taxa at the genus level, providing an avenue for testing more diverse microorganism cells to potentially discover novel microorganism strains having unique properties from different ecosystems.

## Data Availability

Raw sequences of 16S rRNA amplicon genes are available in the NCBI Bioproject database (www.ncbi.nlm.nih.gov/bioproject) under accession number PRJNA1202208.

## SUPPLEMENTAL MATERIAL

Table S1 aem.01794-24-s0001.docx Unique genera from the unencapsulated and encapsulated cultures in three culture media.

ASM does not own the copyrights to Supplemental Material that may be linked to, or accessed through, an article. The authors have granted ASM a non-exclusive, world-wide license to publish the Supplemental Material files. Please contact the corresponding author directly for reuse.
