# Supplementary material for: Microfluidic droplets with amended culture media cultivate a greater diversity of soil microorganisms
Source: Appl Environ Microbiol. 2025 Feb 12;91(3):e01794-24. doi: 10.1128/aem.01794-24 (PMC11921321; doi:10.1128/aem.01794-24)
Supplement: Table S1 — Unique genera from the unencapsulated and encapsulated cultures in three culture media. [file aem.01794-24-s0001.docx]

Supporting Information

**Microfluidic Droplets with Amended Culture Media Cultivate a Greater Diversity of Soil Microogranisms**

Jing Dai^a,†, #^, Yang Ouyang^b, ‡,#^, Rohit Gupte^c^, Xiao Jun A. Liu^b^, Yuwen Li^a^, Fang Yang^d^, Shaorong Chen^e^, Tony Provin^f^, Erin van Schaik^e^, James E. Samuel^e^, Arul Jayaraman^c,d^, Aifen Zhou^b,§^, Paul de Figueiredo^g,h,i*^, Jizhong Zhou^b,j,k,l,m*^, Arum Han^a,c,d*^

^a^ Department of Electrical and Computer Engineering, Texas A&M University, College Station, TX 77843, USA

^b^ Institute for Environmental Genomics, University of Oklahoma, Norman, OK 73019, USA

^c^ Department of Biomedical Engineering, Texas A&M University, College Station, TX 77843, USA

^d^ Department of Chemical Engineering, Texas A&M University, College Station, TX 77843, USA

^e^ Department of Microbial Pathogenesis and Immunology, Texas A&M Health Science Center, Bryan, TX 77807, USA

^f^ Department of Soil and Crop Sciences, Texas A&M University, College Station, TX 77843, USA

^g^ Christopher S Bond Life Sciences Center, University of Missouri, Columbia MO 65211, USA

^h^ Department of Molecular Microbiology and Immunology, University of Missouri School of Medicine, Columbia MO 65211, USA

^i^ Department of Veterinary Pathobiology, University of Missouri School of Veterinary Medicine, Columbia MO 65211, USA

^j^ School of Biological Sciences, University of Oklahoma, Norman, OK 73019, USA

^k^ School of Civil Engineering and Environmental Sciences, University of Oklahoma, Norman, OK 73019, USA

^l^ School of Computer Sciences, University of Oklahoma, Norman, OK 73019, USA

^m^ Earth and Environmental Sciences, Lawrence Berkeley National Laboratory, Berkeley, CA 94720, USA

^†^ Present address: Cytonome, Lexington, MA 02421, USA

^‡^ Present address: Nutrien Ag Solutions, Kerman, CA 93630, USA

^§^ Present address: Department of Chemical Engineering, Texas A&M University, College Station, TX 77843, USA

# These authors contributed equally.

*Co-corresponding authors

Arum Han, arum.han@ece.tamu.edu

Jizhong Zhou, jzhou@ou.edu

Paul de Figueiredo, paullifescience@missouri.edu

Table of content

**Table S1** Unique genera from unencapsulated and encapsulated culture in three culture media

**Table S1** Unique genera from unencapsulated and encapsulated culture in three culture media
